# Effects of Flavonoid Supplementation on Athletic Performance in Healthy Adults: A Systematic Review and Meta-Analysis

**DOI:** 10.3390/nu15214547

**Published:** 2023-10-26

**Authors:** Ying Wang, Zhuang Tian, Zhenyu Li, Jae Cheol Kim

**Affiliations:** Department of Sport Science, Jeonbuk National University, Jeonju 54896, Republic of Korea; wangying890922-@jbnu.ac.kr (Y.W.); 202055434@jbnu.ac.kr (Z.T.); 202055168@jbnu.ac.kr (Z.L.)

**Keywords:** flavonoid supplementation, athletic performance, performance tests, exercise tolerance

## Abstract

Flavonoids, known for their antioxidant properties, can prevent reactive oxygen species (ROS) and influence athletic performance through various physiological and metabolic mechanisms. However, there are conflicting results after summarizing and analyzing the relevant literature. Hence, it is warranted to evaluate the overall impact of flavonoids on athletic performance in healthy adults based on a comprehensive and systematic review and meta-analysis. After searching four databases for literature published since their respective establishments until February 2023 and conducting publication bias and quality assessments, a total of 22 studies were ultimately included. The names and doses of flavonoids, various outcome measurements, as well as types of training, were extracted from included studies. The athletic performance outcomes from the included studies were categorized into ’performance tests’ and ’exercise tolerance,’ depending on the type of training undertaken. Several statistical results, such as pooled effect size (ES), among others, were implemented by meta-analysis using the random effects model. The results of meta-analysis suggest that there is currently sufficient evidence (ES = −0.28; 95% confidence interval (CI): [−0.50, −0.07]; *p* = 0.01 and ES = 0.23; 95% CI: [0.07, 0.39]; *p* = 0.005) to support the notion that flavonoid supplementation enhanced athletic performance in performance tests and exercise tolerance. In addition, among the subgroups, nonsignificant results were observed for athletes (*p* = 0.28) and acute supplementation (*p* = 0.41) in performance tests, as well as athletes (*p* = 0.57) and acute supplementation (*p* = 0.44) in exercise tolerance. Meanwhile, significant results were found for non-athletes (*p* = 0.04) and long-term supplementation (*p* = 0.02) in performance tests, as well as non-athletes (*p* = 0.005) in performance tests and long-term supplementation (*p* = 0.006) in exercise tolerance. The nonsignificant results were likely due to the limitation in the number of related papers, sample sizes, optimal dosage, duration, type of flavonoids, and other factors. Therefore, future research should focus on further investigating these relationships with larger sample sizes, optimal dosage, duration, and type of flavonoids to provide more robust conclusions.

## 1. Introduction

Flavonoids are an important class of natural products extracted from plants, which belong to plant secondary metabolites with polyphenol structure [1]. They have been found in large quantities in vegetables, cocoa, wine, and other plant-based foods as well as beverages; therefore, they are also known as dietary flavonoids [2,3]. The intake of dietary flavonoids has a favorable effect on the prevention and treatment of several diseases due to the antioxidant abilities of flavonoids [4,5,6,7,8,9]. These grounds suggest that flavonoid supplementation could potentially have a positive impact on athletic performance. Certain attributes of flavonoids, like their antioxidant properties, contribute to safeguarding against oxidative stress linked with physical activities and exercise [7]. Therefore, extensive intervention studies have been carried out to investigate whether flavonoid supplementation produces a positive effect on athletic performance. However, to date, related trials of exercise training with flavonoid supplementation have produced equivocal findings.

The beneficial impacts of flavonoids on athletic performance have been affirmed by a substantial amount of research [10,11,12,13,14,15,16,17,18,19,20,21,22,23,24,25]. Cook et al. [19] studied 14 well-trained cyclists who were supplemented with New Zealand black currant (NZBC) extract (105 mg·day^−1^ anthocyanins) for 7 days and improved their performance in a 16.1 km TT by 2.4%. The core reason might be that vasodilation induced by anthocyanin was able to increase the peripheral blood flow in humans to affect the athlete performance. Moreover, the findings discovered by Davis et al. [20] revealed that the intake of quercetin for 7 days increased cycling time when quercetin had induced mitochondrial biogenesis. Additionally, flavonoid supplementation (such as hesperidin [15], blueberry [18], green tea extract [17,21], montmorency cherry [22,23], grape juice [24], pomegranate extract [25], etc.) promoted athlete performance due to its protective role against muscle damage and oxidative stress caused by exercise.

In contrast, in a separate study by García-Merino et al. [26], participants engaged in a 1 km running TT after undergoing 5 g·day^−1^ cocoa supplementation with 425 g flavanols for 10 weeks. Surprisingly, the study did not reveal any performance improvements linked to cocoa supplementation. In addition, Askari et al.’s [27] findings indicated that the intake of quercetin and vitamin C for 8 weeks was not able to achieve the desired mitochondrial changes or demonstrate the biological effects of quercetin in the human body. These studies did not provide support for the enhancement of athletic performance through flavonoid supplementation. It was notable that several other research studies, such as Abbey et al. [28], Brandenburg et al. [29], Dean et al. [30], and Decroix et al. [31], claimed there were insignificant differences on flavonoid supplementation for athletic performance as well.

Hence, it is necessary to review the literature related to flavonoid supplementation in exercise trials to evaluate their overall impacts on athletic performance and to inspire the development of relevant studies. However, to the best of our knowledge, there has been only one paper [32] that conducted a relevant survey on exercise training combined with flavonoids. This systematic review by Ruiz-Iglesias et al. reported that flavonoid supplementation found promising results. Still, no definitive conclusions could be drawn on whether enhancing athletic performance, and the study focused on changes in immune system and inflammatory biomarkers. In summary, we aim to systematically review the effects of flavonoid supplementation on athletic performance. Athletic performance is categorized into performance tests and exercise tolerance. Performance tests include time-trial (TT) [33], while exercise tolerance encompasses time-to-exhaustion tests (TTE) [34] and graded exercise tests (GXT) [35]. Unlike Ruiz-Iglesias et al. [32], this article carries out an overall evaluation using systematic review and meta-analysis. We also investigate the impacts of flavonoid supplementation on training subjects and supplementation duration.

## 2. Methods

The systematic review and meta-analysis were conducted based on the Cochrane Handbook to assess the impacts of flavonoid supplementation. Concretely, this procedure was comprised of three phases. Firstly, we formulated the literature search strategies following the PRISMA^®^ [36] and the PICOS model (see Table 1) [37]. We also established inclusion criteria, after which we preliminarily obtained some literature. Secondly, the publication bias and quality assessment of the included literature were carried out to exclude inconsistent literature. Finally, we undertook data extraction and statistical analysis. Moreover, this meta-analysis has been registered in PROSPERO (Registration number: CRD42023425112) before starting this work.

### 2.1. Literature Search Strategies

The two authors (Y.W. and Z.T.) conducted literature searches on PubMed, Web of Science (WOS), Cochrane Library, and Embase. All full-text literature published in English since the establishment of each database until February 2023 were reviewed. The used Boolean searching equation included: “Flavonoids” [Title/Abstract] OR “Flavonoid” [Title/Abstract] OR “Bioflavonoids” AND “Athletic Performance” [Mesh] OR “Performance, Athletic” [Title/Abstract] OR “Sports Performance” [Title/Abstract] AND “randomized” [tiab] OR “placebo” [tiab] OR “randomly” [tiab] OR “trial” [tiab] OR “groups” [tiab] OR “randomized controlled trial” [pt] OR “controlled clinical trial” [pt]. Meanwhile, a comprehensive manual search of relevant publications, including journal articles and reference lists, was performed to fully ensure the most comprehensive inclusion of pertinent studies.

### 2.2. Inclusion Criteria

The inclusion criteria of studies were as follows:

- Studies were carried out on healthy adults without disease;
- Studies for which the experiments must contain randomized controlled trial;
- Studies for which the full text must be English.

### 2.3. Searching Procedure

**Figure 1:**
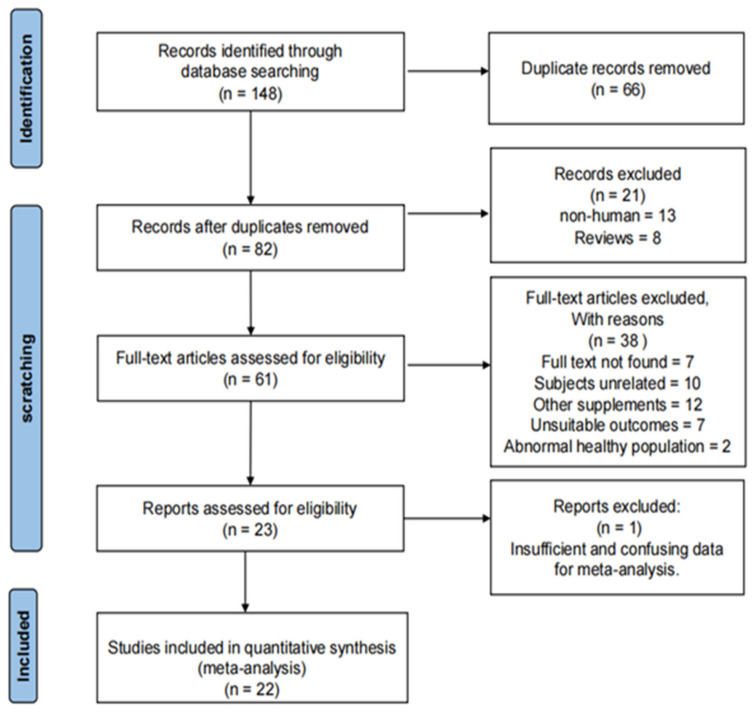
The flow diagram of searching strategy.

The selection procedure of included literature was shown in Figure 1. The main procedures were as follows:

- 148 records were obtained from specified four databases;
- After removing 66 duplicate records, 82 articles remained, from which we removed eight reviews and 13 non-human experimental studies again;
- A total of 61 articles met the eligibility criteria, and their full texts were assessed;
- After finishing a comprehensive and systematic evaluation, 38 articles were excluded, seven for which full texts were not found, 10 on unrelated subjects, 12 included another supplements, seven had unsuitable outcomes, two used abnormal healthy populations and one had insufficient data, respectively;
- Finally, a total of 22 studies were included for the meta-analysis.

### 2.4. Publication Bias

Excluding literature with negative results might lead to a biased outcome in the meta-analysis; thus, we assessed publication bias for all included literature. The funnel plots for visual interpretation and Egger’s statistics were conducted to confirm the presence of publication bias.

### 2.5. Quality Assessment of the Experiments

The quality and interpretation of the research were determined by two independent reviewers (Y.W. and Z.T.) in accordance with the Cochrane Collaboration Guidelines [37,38] using Review Manager 5.3 software. The checklist areas covered seven criteria of quality assessment.

### 2.6. Data Extraction

All relevant trial data were obtained in electronic databases and screened independently by two authors (Y.W. and Z.T.). If there was a disagreement, it was necessary to discuss or have a third-party arbitration (Z.Y.L.). From the included studies, we extracted key details such as the first author, year of publication, study type, subjects’ gender, flavonoid name and dose, administration method and timing, control group regimen, training type, and outcome measures, including sample size and mean ± standard deviation (SD). If some included studies contained more than one supplement intervention or multiple athletic output indicators, they needed to be coded as separate results according to multi-intervention and multi-output studies.

### 2.7. Statistical Analysis

The sample size and mean ± SD extracted from included literature were employed to conduct the meta-analysis. If the outcome measures included standard error (SE) in the extracted data, SD was calculated as SD = SE × $\sqrt{n}$ [39], where *n* was the sample size.

The standardized mean difference (SMD) involved the use of mean ± SD from experimental and control groups to standardize the difference in means, while the Effect Size (ES) was typically the weighted average of the SMD. Both were statistical measures used to quantify the magnitude of the difference between experimental and control groups. In our study, the SMD was calculated using Hedges’ g and it was used as an estimate for the effect size. Therefore, in this context, SMD and ES were equivalent [40,41,42]. The evaluation criteria of ES followed Cohen criteria: <0.2, trivial; 0.2–0.5, small; 0.5–0.8, moderate; and >0.8, large [43]. It was notable that a negative ES for a performance variable suggested an ergogenic effect. The statistics *I*^2^ indicated the degree of heterogeneity between studies [37]: 25–50%, small inconsistency; 50–75%, moderate inconsistency; and >75%, high inconsistency [43].

We used the forest plot to present the overall analysis results. If the *p* ≤ 0.05, 95% CI without 0, then it was statistically significant.

## 3. Results

### 3.1. Publication Bias

**Figure 2:**
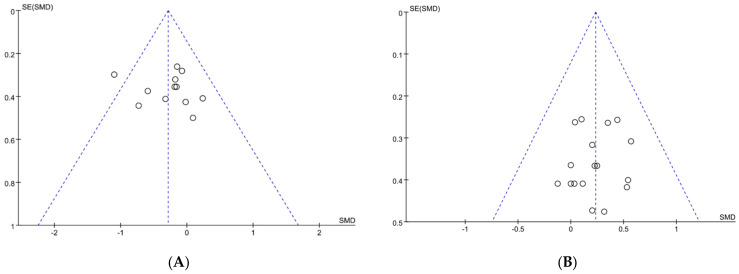
Funnel plot: publication bias risk in performance test (**A**) and in exercise tolerance (**B**). Where both slanting dotted lines represent the expected 95% CI; vertical dotted line refer to the position of no effect; circles represent individual studies.

As shown in Figure 2, a standard funnel plot was made to assess publication bias. We found that the literature on exercise tolerance showed overall good symmetry, while the literature on performance tests exhibited some degree of asymmetry. Additionally, one study, Nieman et al. [44], surpassed the boundary of funnel plot. So, to further validate the potential presence of publication bias, an assessment was conducted using Egger’s statistical test. As a result, we found no publication bias for performance tests and exercise tolerance with an observed significance level *p* > 0.05 [45]. It is worth noting that the article by Nieman et al. did not have a bias risk and should be included in our analysis [44]. To sum up, publication bias was not presented in our included literature, all the papers could be used to conduct the meta-analysis.

### 3.2. Quality Assessment of the Experiments

**Figure 3:**
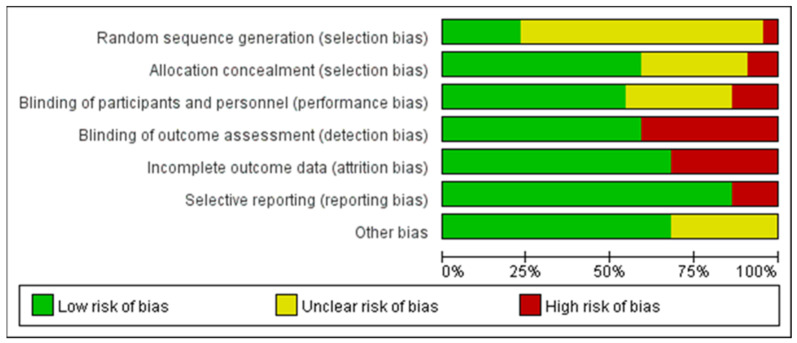
Graph of risk of bias.

**Figure 4:**
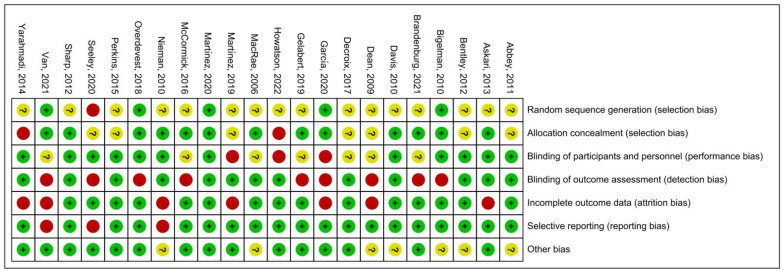
Summary of risk of bias [11,20,26,27,28,29,30,31,44,45,46,47,48,49,50,51,52,53,54,55,56,57,58]. Where the “green +” represents low risk of bias; “yellow ?” represents unclear risk of bias; “red -” represents high risk of bias.

The quality assessment of the experiments was conducted based on the “Cochrane Collaboration Guidelines for Assessing Quality in Experiments” [37,38]. This evaluation encompassed seven vital criteria: ensuring participants’ random assignment (Random Sequence Generation); maintaining the concealment of trial group allocation (Allocation Concealment); making certain both participants and personnel are unaware of group assignments (Blinding of Participants and Personnel); verifying that those assessing outcomes are blinded to group designations (Blinding of Outcome Assessment); addressing any potential gaps in trial data (Incomplete Outcome Data); ensuring all study results, whether favorable or not, are reported (Selective Reporting); and considering any other possible biases (Other Bias). The results are shown in Figure 3, where each item was expressed as a percentage relative to the total number of studies included. We found that there was an unclear risk of bias on random sequence generation due to 16 papers out of 22 being ambiguous about their random assignment methods. In addition, the remaining six factors of quality assessment were identified as low risk. Meanwhile, Figure 4 presented the risk of bias for each study as well. Hence, we believed the quality assessment of the experiments of all included studies presented a low risk. Therefore, we were able to proceed with a meta-analysis.

### 3.3. Study Characteristics

Based on the methodology of Section 2, the participants and intervention characteristics of 22 studies are shown in Table 2, which illustrates subject, experimental design, intervention, exercise protocol, outcomes, trial results (means ± SD), and main conclusion of each study. We noted from Table 2 that the effects of flavonoid supplementation on athletic performance varied across different studies, indicating a lack of consensus. Given this variability and the challenges in visually discerning any consistent impact, a thorough and systematic meta-analysis becomes imperative, especially for performance tests and exercise tolerance.

### 3.4. Performing a Meta-Analysis on Performance Tests

**Figure 5:**
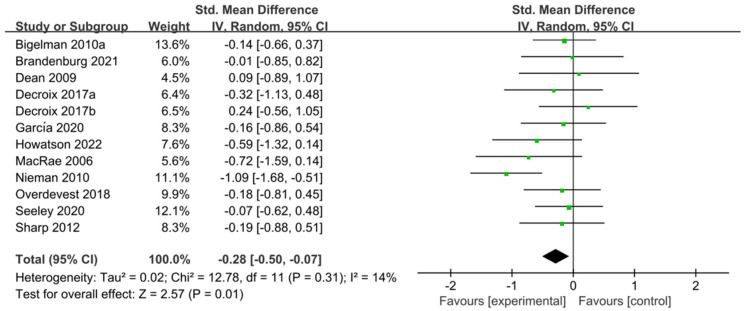
Forest plot: meta-analysis of flavonoid impacts on performance tests [26,29,30,31,44,47,48,49,53,55,56]. Where each color square represents the point estimate of the ES (SMD); black diamond represents the combined or pooled effect size and its 95% CI from all the studies.

The 11 studies [5,29,30,31,44,47,48,49,53,55,56] related to TT were included in the meta-analysis of performance tests; these 11 studies contain 401 participants and 12 ESs in total. Figure 5 indicated that flavonoid supplementation presented a significant effect on the performance tests derived from the TT regimen.

Although a significant effect had been observed in healthy adults based on the above discussion, we still would like to investigate the effects of flavonoids on both athletes and non-athletes, as well as examine the impact of acute supplementation and long-term supplementation duration. In this study, the distinction between athletes and non-athletes is determined by whether they have undergone systematic professional training. Additionally, the categorization between acute supplementation and long-term supplementation is made based on the duration of flavonoid supplementation. Specifically, if the supplementation lasts for more than 7 days, it is viewed as long-term supplementation, while anything less is regarded as acute supplementation [59].

**Figure 6:**
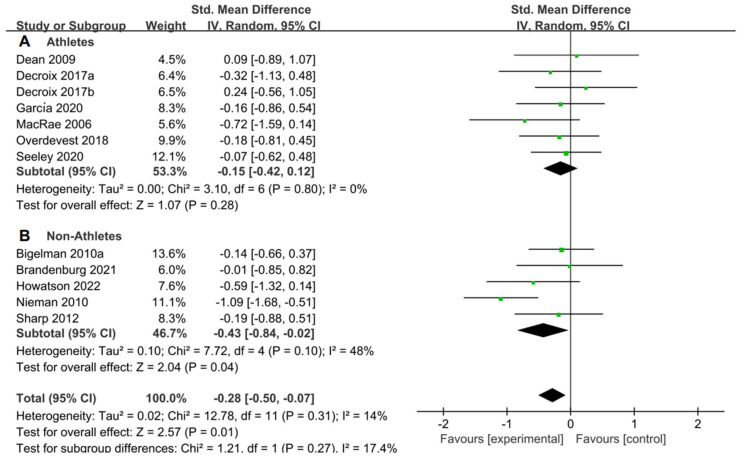
Forest plot: athlete and non-athlete subgroup analysis of flavonoid impacts on performance tests. (**A**) Athlete subgroup. (**B**) Non-athlete subgroup [26,29,30,31,44,47,48,49,53,55,56].

For the subgroup analysis of athletes and non-athletes, Figure 6A suggested that flavonoid supplementation revealed no effect on performance tests in athletes. Conversely, as shown in Figure 6B, flavonoid supplementation presented a significant effect on the performance tests in non-athletes.

**Figure 7:**
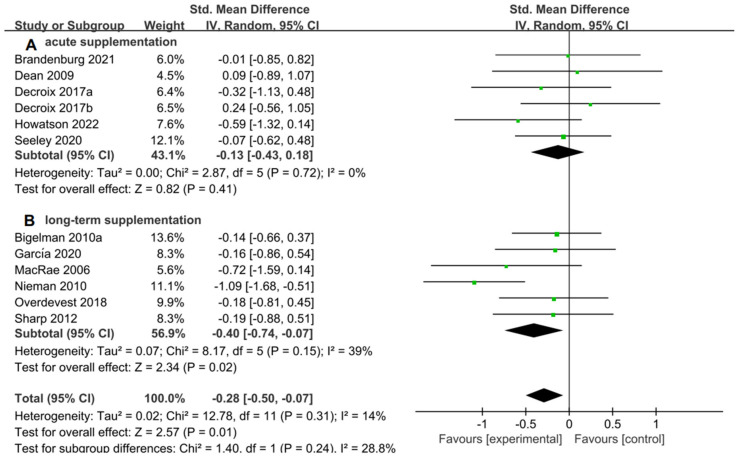
Forest plot: acute and long-term supplementation subgroup analysis of flavonoid impacts on performance tests. (**A**) Acute supplementation subgroup. (**B**) Long-term supplementation subgroup [26,29,30,31,44,47,48,49,53,55,56].

For the subgroup analysis of acute supplementation and long-term supplementation. Figure 7A suggested that flavonoid supplementation revealed no effect on performance tests in acute supplementation. However, as shown in Figure 7B, flavonoid supplementation presented a significant effect on the performance tests when long-term supplementation duration was conducted.

### 3.5. Performing a Meta-Analysis on Exercise Tolerance

**Figure 8:**
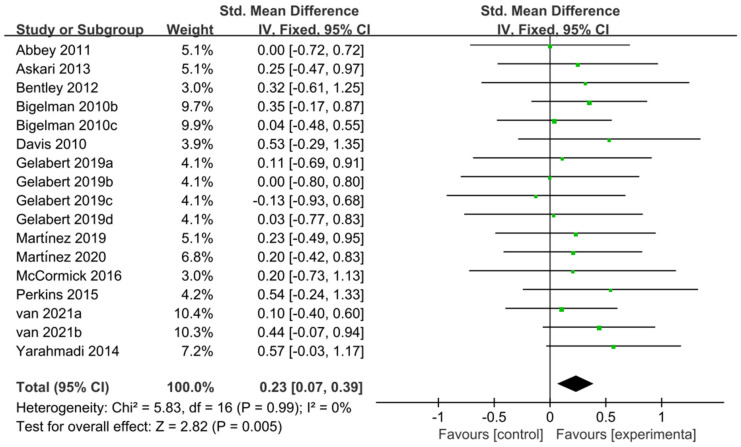
Forest plot: meta-analysis of flavonoid impacts on exercise tolerance [11,20,27,28,46,47,50,51,52,54,57,58].

The 12 studies [11,20,27,28,46,47,50,51,52,54,57,58] related to TTE and GXT were used to perform the meta-analysis of exercise tolerance; these 12 studies contain 595 participants and 17 ESs in total. Figure 8 indicated that flavonoid supplementation presented a significant effect on the exercise tolerance derived from TT regimen.

Although a significant effect had been observed in healthy adults based on the above discussion, we still would like to investigate the effects of flavonoids on both athletes and non-athletes, as well as examine the impact of acute supplementation and long-term supplementation duration.

**Figure 9:**
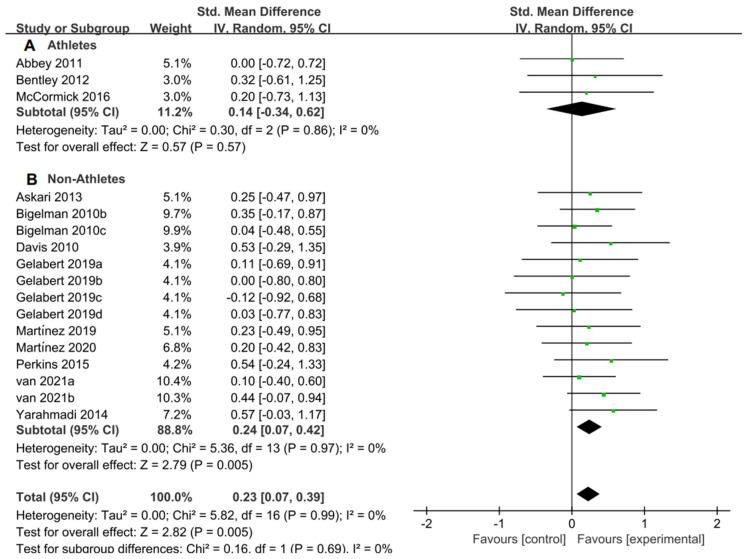
Forest plot: athlete and non-athlete subgroup analysis of flavonoid impacts on exercise tolerance. (**A**) Athlete subgroup. (**B**) Non-athlete subgroup [11,20,27,28,46,47,50,51,52,54,57,58].

For the subgroup analysis of athletes and non-athletes, Figure 9A suggested that flavonoid supplementation revealed no effect on exercise tolerance in athletes. Conversely, as shown in Figure 9B, flavonoid supplementation presented a significant effect on exercise tolerance in non-athletes.

**Figure 10:**
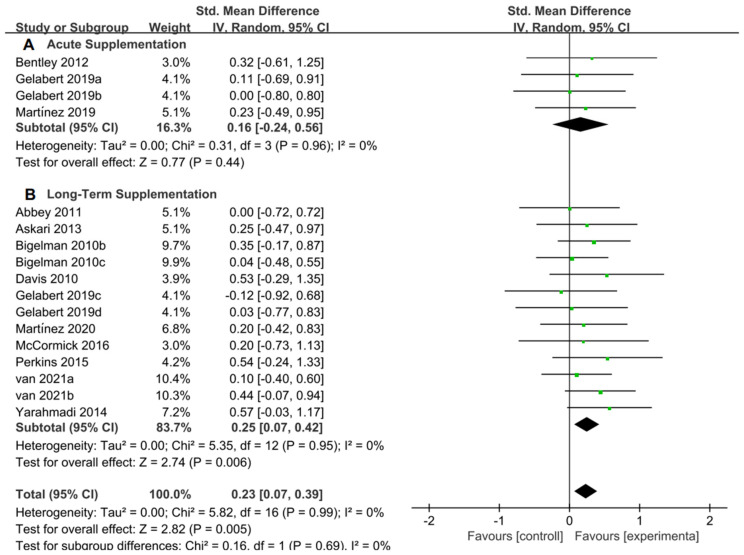
Forest plot: acute and long-term supplementation subgroup analysis of flavonoid impacts on exercise tolerance. (**A**) Acute supplementation subgroup. (**B**) Long-term supplementation subgroup [11,20,27,28,46,47,50,51,52,54,57,58].

For the subgroup analysis of acute supplementation and long-term supplementation, Figure 10A suggested that flavonoid supplementation revealed no effect on exercise tolerance in acute supplementation. However, as shown in Figure 10B, flavonoid supplementation presented a significant effect on exercise tolerance, when long-term supplementation duration was conducted.

## 4. Discussion

A significant difference in performance tests had been identified when the subject had a specified dosage of flavonoid supplementation in TT regimen by meta-analysis. Based on the findings of the meta-analysis of performance tests, we firmly believed that flavonoid supplementation might contribute to slight variations in athletic performance by inducing distinct metabolic pathways or a regulation of mitochondrial biogenesis, which could result in significant differences in performance tests. Accordingly, our statements also align with the reasons for which flavonoid supplementation has produced a significant effect on performance tests in this literature [44,49,55]. However, no significant changes in performance tests were observed in certain studies, as evidenced by references [26,29,30,31,47,56]; however, we must emphasize that there might be explainable reasons behind their conclusions. The excessive intensity of exercise and the dosage of the supplement could inhibit mitochondrial biogenesis and function while suppressing excessive oxidative stress. Therefore, flavonoid supplementation can improve performance tests, but the intensity of exercise and dosage, type, as well as duration of the supplement must be appropriate [32]. Too little dosage might present non-\significance, while too much could produce a negative effect. Specific dosages might be a better choice. In addition, different flavonoids have unique impacts as well. Meanwhile, different intake duration could induce different performance changes; for example, quercetin (1000 mg/day, 2 weeks) [44], anthocyanin (150 mg/day, 6 day) [48], vitamins and quercetin (600 mg/day, 6 weeks) [49] citrus flavonoid (500 mg/day, 4 weeks) [53], and soy (30 g, once) [55] improved performance.

Results from the athlete and non-athlete subgroup analysis suggested that the exercise subjects’ characteristics might influence the efficacy of flavonoid supplementation in performance tests. We observed significant results in non-athletes but not in athletes, which might be attributed to exercise subjects’ characteristics. García-Merino et al. [26] reported a statistically insignificant improvement increased by 1.27% after athlete intake flavonoid supplementation in a TT experiment. However, Overdevest et al. [53] found a statistically significant improvement increased by 5% after non-athletes underwent 500 mg·day^−1^ hesperidin supplementation for 4 weeks. It is noteworthy that non-athletes have greater room for improvement compared to athletes. The reason revealed is also consistent with the conclusion discussed by Hopkins et al. in [60]. Therefore, the exercise subjects’ characteristics should be emphasized.

There was a significant improvement for performance tests with long-term supplementation, but for acute supplementation, it was insignificant. These could be related to the duration of supplementation, because the long-term could result in an increase in muscle-oxidation [61,62]. Decroix et al. [31] found that acute supplementation of cocoa flavanols (CF) could marginally enhance the relative power output following TT exercise, and Dean et al. [30] carried out acute supplementation with 270 mg of green tea extract. This acute supplementation did not produce improvements. However, Wolfram et al. [63] observed that undergoing green tea supplementation beyond 12 weeks might yield more beneficial effects in human experiments.

A significant difference of exercise tolerance has been identified when the subject intake specified dosage flavonoid supplementation in TTE or GXT regimen by meta-analysis. As we all know, flavonoids boost exercise tolerance by amplifying oxygen delivery to muscles, increasing nitric oxide (NO) concentrations and strengthening antioxidant defenses. For example, Van Iersel et al. [57] carried out the daily intake of 400 mg/day of citrus flavonoids over 8 weeks, resulting in a substantial enhancement in the capacity to supply oxygen to muscles. This improvement was achieved through the elevation of NO levels, ultimately leading to increased average power output in the Wingate test. Furthermore, Yarahmadi, et al. [58] conducted the Bruce exercise test after 6 consecutive weeks of supplementing the subjects with 100 mg of anthocyanins per day, indicated a significant improvement in Vo_2_ max due to attributing to the antioxidant properties exhibited by anthocyanins. This coincides perfectly with our findings. Meanwhile, we must consider the effects of dosage, type, and duration of flavonoid supplementation as well as exercise subjects’ characteristics too. Proper dosing can extend aerobic endurance and reduce recovery time. Some benefit cardiovascular endurance, others aid muscle recovery; for example, luteolin and mangiferin (50 mg and 140 mg for 48 h) [11], luteolin and mangiferin (100 mg and 420 mg for 15 days) [11], lactaway (150 mL for once) [46], quercetin (1000 mg/day, 7 days) [20], 2S-hesperidin (500 mg for once) [50], 2S-hesperidin (500 mg/day, 8 weeks) [51], New Zealand black currant (300 mg/day, 7 days) [54], citrus flavonoid (400 mg or 500 mg/day, 4 weeks) [57], and anthocyanin (100 mg/day, 6 weeks) [58] improved performance.

The results from the athlete and non-athlete subgroup analysis indicated that the exercise subjects’ characteristics might influence the efficacy of flavonoid supplementation on exercise tolerance as well. Athletes, due to their prolonged, high-intensity training, have developed robust adaptive responses, particularly in areas of antioxidant activity, energy metabolism, and inflammatory reactions. When they supplement with flavonoids, these compounds may further amplify these established adaptive mechanisms. In contrast, non-athletes might not have such pronounced adaptive responses. This difference in adaptive reactions explains why athletes and non-athletes may exhibit distinct endurance performance outcomes after flavonoid supplementation. Well-trained athletes showcase enhanced physiological adaptations due to consistent training, particularly in areas like antioxidant activity, mitochondrial function, and NO production [20,64]. While athletes already exhibit superior antioxidative and mitochondrial capacities, non-athletes can achieve more pronounced performance boosts from quercetin supplementation [54]. Furthermore, black currant intake in healthy adults can delay muscle fatigue and combat exercise-induced ROS. Such benefits are largely attributed to the role of exercise in activating eNOS, promoting NO synthesis, and enhancing blood flow [65]. Therefore, supplementation with flavonoids has been proven effective for non-athletes, but its impact on athletes still warrants further research. Moreover, adaptability and the characteristics of the subjects should be taken into account.

In addition, regarding acute supplementation and long-term supplementation in exercise tolerance. Long-term supplementation with flavonoids may enhance endurance by improving blood flow, strengthening antioxidant defenses, alleviating chronic inflammation, and optimizing energy metabolism. In contrast, acute supplementation might offer immediate vasodilation, short-term antioxidant protection, and rapid alleviation of inflammatory responses. As noted by Van Iersel et al. [57], the citrus flavonoid group could enhance athletes’ anaerobic capacity after 8 weeks by upregulating NO, scavenging ROS, or regulating mitochondrial biogenesis. Gelabert-Rebato et al. [11] declared that peak power output exhibited a significant increase whether it is a long-term supplementation or an acute supplementation after oral mangiferin and luteolin supplementation. There were only three studies of acute flavonoid supplementation, which could result in the increasing risk of inaccurate estimates. Therefore, it is imperative to exercise prudence in interpreting the findings, while taking into account the constraints posed by the limited scope and number of studies included in the analysis.

Finally, in view of the limitations of exercise regimens, sample size, flavonoid intake, diverse designs and target population led to unstable statistical results, so, further research was necessary to establish much more incontrovertible evidence regarding exercise regimens. While many of the articles included in the analysis focused on evaluating athletic performance, a subset of studies only reported it as a secondary outcome. As a result, a comprehensive and thorough evaluation of athletic performance cannot be conducted. In terms of sample size, in clinical studies, experimental subjects were often limited by various factors such as specific populations, time constraints, and resource availability, leading to insufficient sample sizes and hindering more in-depth analyses. Concerning the number of relevant studies, the fewer related research studies result in an unstable statistical result; for example, there were only three studies for acute flavonoid supplementation on acute supplementation subgroup of exercise tolerance, but at least two studies were required to perform a meta-analysis [35]. Fewer studies might increase the risk of inaccurate estimates and limit the generalizability of the results. Thereby, the exercise regimens, sample size, and the number of correlated research were challenges in conducting systematic review and meta-analysis, these challenges provided opportunities for our future research endeavors and underscore the necessity for further investigations in this field. Future studies on flavonoid supplementation and athletic performance should consider diverse designs, notably randomized controlled trials and dose-response trials, while targeting varied populations, including elite vs. recreational athletes, different age groups, and sport-specific groups. It is essential to control for confounding factors such as diet, training load, and other supplements to ensure accurate findings.

In summary, flavonoid supplementation had a significant improvement in performance tests using the TT regimen. Meanwhile, the subgroup analysis, athletes and non-athletes as well as for acute supplementation and long-term supplementation, was carried out, and found that supplementing flavonoid is slightly beneficial for non-athlete and long-term supplementation, whereas it was not useful for athlete and acute supplementation. However, it was essential to conduct further research to solve some limitations. Additionally, flavonoid supplementation demonstrated that there was a modest yet statistically significant ES for exercise tolerance using the TTE and GXT regimens as well. Subsequently, we performed further subgroup analyses on exercise tolerance too. Results indicated that the non-athletes group presented a significant improvement, but there was no significance in athletes group. Meanwhile, long-term supplementation was found to effectively enhance exercise tolerance, whereas no changes were observed with acute supplementation. Consequently, athletic performance would present a significant improvement in exercise tolerance when non-athletes took flavonoid supplementation for a long-term. However, much further research with a larger sample size was essential for athletes and acute supplementation.

## 5. Conclusions

In summary, our meta-analysis results indicate that flavonoid compounds can provide positive support for the athletic performance of adults. Particularly in the cases of non-athletes and/or long-term supplementation, flavonoid supplementation is effective in enhancing athletic performance. However, factors such as different training environments, variations in supplement dosage, and duration of intake can influence its effects. Thus, further research is needed to establish more concrete evidence regarding the impact of flavonoid supplementation on athletic performance.

## Figures and Tables

**Table 1:** PICOS criteria used to perform the systematic review.

PICOS	Standard
Population Intervention	Healthy adults Flavonoid supplementation
Comparator	Placebo
Outcome	Athletic performance
Study design	Randomized controlled trial (RCT)

**Table 2:** Participants and intervention characteristics.

Reference	Subject, *n*	Experimental Design	Intervention	Exercise Protocol	Outcomes	Trial Results (Mean ± SD)	Main Conclusion
Abbey, 2011 [28]	Athletes, *n* = 15 (males)	Double-blinded, cross-over, RCT	1000 mg Quercetin a day for 7 days	12 × 30-m sprints	Average times	Q = 4.85 ± 0.24 s *P* = 4.85 ± 0.24 s	→
Askari, 2013 [27]	Students with athletic history 3 years, *n* = 60 (males)	Double-blind, parallel, RCT	500 mg Quercetin & 250 mg Vitamin C 500 mg a day for 8 weeks	Bruce protocol	Total distance	Q = 1289.07 ± 221.74 m *P* = 1233.92 ± 209.85 m	→
Bentley, 2012 [46]	Cyclists, *n* = 9 (males)	Double-blind, RCT	150 mL Lactaway for once	Time to exhaustion	Total time	LA = 574 ± 265 s *P* = 494 ± 213 s	↑
Bigelman, 2010 [47]	Moderately trained person, *n* = 58 (44 males and 14 females)	Double-blind, parallel, RCT	1000 mg Quercetin a day for 42–54 days	2-mile running TT, Wingate anaerobic test, 36.6-m Sprint	Total time Mean power Total time	Q = 14.92 ± 1.74 min *P* = 15.17 ± 1.67 min (a)	→
						Q = 551.72 ±110.99 W *P* = 513.21 ± 104.98 W (b)	→
						Q = 5.78 ± 0.50 s *P* = 5.76 ± 0.52 s (c)	→
Brandenburg, 2021 [29]	Recreational runners, *n* = 11 (4 males and 7 females)	Double-blinded, cross-over, RCT	324 g Blueberry a day for 4 days	30 min TT in norm baric hypoxia	Total distance	BLU = 4.47 ± 0.69 km *P* = 4.46 ± 0.62 km	→
Davis, 2010 [20]	Student volunteers, *n* = 12 (7 males and 5 females)	Double-blinded, cross-over, RCT	1000 mg Quercetin a day for 7 days	Endurance	Ride time	Q = 105.7 ± 21.8 min *P* = 93.4 ± 22.9 min	↑
Dean, 2009 [30]	Well-trained cyclists, *n* = 10 (males)	Double-blinded, cross-over, RCT	270 mg Green-tea extract (TEAVIGO) a day for 5 days and 270 × 2 on Day 6	40-km Cycling TT	Total time	GT = 3652 ± 295 s *P* = 3627 ± 211 s	→
Decroix, 2017 [31]	Cyclists, *n* = 12 (male)	Double-blind, cross-over, RCT	900 mg Cocoa Flavanols for once	Two 30 min running TT	Total time	CO = 29.22± 1.32 min *P* = 29.78 ± 1.97 min (a)	→
						CO = 30.0 ± 1.58 min *P* = 29.57 ± 1.82 min (b)	→
García, 2020 [26]	Endurance cross-country athletes, *n* = 44 (males)	Blind, parallel, RCT	5 g Cocoa a day for 10 weeks (425 mg of flavanols)	1 km Running TT	Total time	CO = 3.15 ± 0.21 min *P* = 3.19 ± 0.27 min	→
Gelabert, 2019 [11]	Physical education students, *n* = 12 (male)	Double-blind, cross-over, RCT	L: 50 mg peanut and 140 mg MLE a day for 48 h and 15 days H: 100 mg peanut and 420 mg MLE a day for 48 h and 15 days	Incremental exercise test	Output power	48 h MA + LU (L) = 288 ± 86.6 W *P* = 277 ± 103.9 W (a)	↑
						MA + LU (H) = 291 ± 166.3 W *P* = 291 ± 166.3 W(b)	→
						15 days MA + LU (L) = 271 ± 83.1 W *P* = 282 ± 86.6 W(c)	→
						MA + LU (H) = 291 ± 162.8 W *P* = 286 ± 145.5 W(d)	↑
Howatson, 2022 [48]	Volunteer, *n* = 30 (male)	Double-blind, parallel, RCT	Haskap (150 mg Anthocyanin) a day for 6 days	5 km Running TT	Total time	Has = 1282 ± 140 s *P* = 1384 ±193 s	↑
MacRae, 2006 [49]	Elite cyclists, *n* = 11 (male)	Double-blind, cross-over, RCT	600 mg Vitamins & Quercetin a day for 6 weeks	30 km Cycling TT	Total time	FRS = 50.70 ± 2.22 min *P* = 52.30 ± 2.03 min	↑
Martínez, 2019 [50]	Amateur cyclists, *n* = 16 (male)	Single-blind, cross-over, RCT	500 mg 2S-hesperidin for once	4 × 30 s all-out sprints	Average power	2S-hes = 567.84 ± 55.44 W *P* = 555.25 ± 51.81 W	↑
Martínez, 2020 [51]	Amateur cyclists, *n* = 40 (male)	Double-blind, parallel, RCT	500 mg 2S-hesperidin a day for 8 weeks	Wingate test	Absolute peak power	2S-hes = 860.6 ± 70.37 W *P* = 840.2 ± 118.93 W	↑
McCormick, 2016 [52]	Water polo players, *n* = 9 (male)	Double-blind, cross-over, RCT	90 mL Cherry juice (9.117 mg/mL of anthocyanins) a day for 7 days	Water polo intermittent swim test	Total distance	CJ = 605 ± 239 m *P* = 558 ± 203 m	→
Nieman, 2010 [44]	Adults, *n* = 26 (male)	Double-blind, cross-over, RCT	1000 mg Quercetin a day for 2 weeks	12 min TT	Total distance	Q = 1013 ± 20.9 m *P* = 990 ± 20.5 m	↑
Overdevest, 2018 [53]	Trained athletes, *n* = 40 (male)	Double-blind, parallel, RCT	500 mg Citrus Flavonoid a day for 4 weeks	10 min TT	Absolut power	CF = 313 ± 43.6 W *P* = 304.3 ± 51 W	↑
Perkins, 2015 [54]	Healthy adults, *n* = 13 (males)	Double-blind, cross-over, RCT	300 mg New Zealand Black Currant a day for 7 days	6 × 19 s of sprints	Total distance	NZBC = 4282 ± 833 m *P* = 3871 ± 622 m	↑
Seeley, 2020 [55]	Cyclists and triathletes, *n* = 25 (male)	Double-blind, cross-over, RCT	30 g Soy for once	20-km TT	Total time	Soy = 35.31 ± 3.11 min *P* = 35.53 ± 3.09 min	↑
Sharp, 2012 [56]	Healthy volunteers, *n* = 16 (male)	Double-blind, cross-over, RCT	1000 mg Quercetin a day for 8.5 day	200-kJ Cycle TT	Total time	Q = 18.3 ± 1.0 min *P* = 18.5 ± 1.1 min	→
Van, 2021 [57]	Moderately trained volunteers, *n* = 93 (males and females)	Double-blind, parallel, RCT	400 mg(a) or 500 mg(b) Citrus Flavonoid extract a day for 4 weeks	Wingate anaerobic test	Average power	CFE = 526 ± 150 W *P* = 511 ± 140 W (a)	↑
						CFE = 575 ± 148 W *P* = 511 ± 140 W (b)	↑
Yarahmadi, 2014 [58]	Athletes, *n* = 54 (22 female and 32 male)	Double-blind, parallel, RCT	100 mg Anthocyanin a day for 6 weeks	Bruce treadmill test	Vo_2_ max	Ant = 52.62 ± 5.04 L/min *P* = 49.61 ± 5.33 L/min	↑

RCT: randomized controlled trial; TT: time trial; *P*: placebo trial, L: group of low-dose; H: group of high-dose; Vo_2_ max: maximal oxygen uptake; ↑: improved athletic performance; →: unimproved athletic performance; (a), (b), (c) and (d) indicate certain paper uses more than one test or supplementation protocol.

## Data Availability

The data presented in this study are available in the inserted articles.

## References

[1] Singh B., Kumar A., Malik A.K. (2017). Flavonoids biosynthesis in plants and its further analysis by capillary electrophoresis. Electrophoresis.

[2] Steinberg F.M., Bearden M.M., Keen C.L. (2003). Cocoa and chocolate flavonoids: Implications for cardiovascular health. J. Am. Diet. Assoc..

[3] Baby J., Devan A.R., Kumar A.R., Gorantla J.N., Nair B., Aishwarya T.S., Nath L.R. (2021). Cogent role of flavonoids as key orchestrators of chemoprevention of hepatocellular carcinoma: A review. J. Food Biochem..

[4] Rupasinghe H.V. (2020). Special Issue “Flavonoids and Their Disease Prevention and Treatment Potential”: Recent Advances and Future Perspectives. Molecules.

[5] García-Barrado M.J., Iglesias-Osma M.C., Pérez-García E., Carrero S., Blanco E.J., Carretero-Hernández M., Carretero J. (2020). Role of flavonoids in the interactions among obesity, inflammation, and autophagy. Pharmaceuticals.

[6] Liskova A., Koklesova L., Samec M., Varghese E., Abotaleb M., Samuel S.M., Smejkal K., Biringer K., Petras M., Blahutova D. (2020). Implications of flavonoids as potential modulators of cancer neovascularity. J. Cancer Res. Clin. Oncol..

[7] Chen Q., Xu B., Huang W., Amrouche A.T., Maurizio B., Simal-Gandara J., Tundis R., Xiao J., Zou L., Lu B. (2020). Edible flowers as functional raw materials: A review on anti-aging properties. Trends Food Sci. Technol..

[8] Li G., Ding K., Qiao Y., Zhang L., Zheng L., Pan T., Zhang L. (2020). Flavonoids Regulate Inflammation and Oxidative Stress in Cancer. Molecules.

[9] Cichon N., Saluk-Bijak J., Gorniak L., Przyslo L., Bijak M. (2020). Flavonoids as a Natural Enhancer of Neuroplasticity—An Overview of the Mechanism of Neurorestorative Action. Antioxidants.

[10] Kressler J., Millard-Stafford M., Warren G.L. (2011). Quercetin and endurance exercise capacity: A systematic review and meta-analysis. Med. Sci. Sports Exerc..

[11] Gelabert-Rebato M., Wiebe J.C., Martin-Rincon M., Galvan-Alvarez V., Curtelin D., Perez-Valera M., Juan Habib J., Pérez-López A., Vega T., Morales-Alamo D. (2019). Enhancement of exercise performance by 48 hours, and 15-day supplementation with magiferin and luteolin in men. Nutrients.

[12] Braakhuis A.J., Somerville V.X., Hurst R.D. (2020). The effect of New Zealand blackcurrant on sport performance and related biomarkers: A systematic review and meta-analysis. J. Int. Soc. Sports Nutr..

[13] Davis J.M., Murphy E.A., Carmichael M.D. (2009). Effects of the Dietary Flavonoid Quercetin Upon Performance and Health. Optom. Vis. Sci..

[14] Braakhuis A.J., Hopkins W.G. (2015). Impact of Dietary Antioxidants on Sport Performance: A Review. Sports Med..

[15] Imperatrice M., Cuijpers I., Troost F.J., Sthijns M.M. (2022). Hesperidin functions as an ergogenic aid by increasing endothelial function and decreasing exerciseinduced oxidative stress and inflammation, thereby contributing to improved exercise performance. Nutrients.

[16] Decroix L., Soares D.D., Meeusen R., Heyman E., Tonoli C. (2018). Cocoa Flavanol Supplementation and Exercise: A Systematic Review. Sports Med..

[17] Jówko E., Sacharuk J., Balasińska B., Ostaszewski P., Charmas M., Charmas R. (2011). Green tea extract supplementation gives protection against exercise-induced oxidative damage in healthy men. Nutr. Res..

[18] Bloedon T., Vendrame S., Bolton J., Lehnhard R., Riso P., Klimis-Zacas D. (2015). The effect of wild blueberry (*Vaccinium angustifolium*) consumption on oxidative stress, inflammation, and DNA damage associated with exercise. Comp. Exerc. Physiol..

[19] Cook M.D., Myers S.D., Blacker S.D., Willems M.E.T. (2015). New Zealand blackcurrant extract improves cycling performance and fat oxidation in cyclists. Eur. J. Appl. Physiol..

[20] Davis J.M., Carlstedt C.J., Chen S., Carmichael M.D., Murphy E.A. (2010). The Dietary Flavonoid Quercetin Increases VO2max and Endurance Capacity. Int. J. Sport Nutr. Exerc. Metab..

[21] Kuo Y.-C., Lin J.-C., Bernard J.R., Liao Y.-H. (2015). Green tea extract supplementation does not hamper endurance-training adaptation but improves antioxidant capacity in sedentary men. Appl. Physiol. Nutr. Metab..

[22] Levers K., Dalton R., Galvan E., O’connor A., Goodenough C., Simbo S., Mertens-Talcott S.U., Rasmussen C., Greenwood M., Riechman S. (2016). Effects of powdered Montmorency tart cherry supplementation on acute endurance exercise performance in aerobically trained individuals. J. Int. Soc. Sports Nutr..

[23] Morgan P.T., Barton M.J., Bowtell J.L. (2019). Montmorency cherry supplementation improves 15-km cycling time-trial performance. Eur. J. Appl. Physiol..

[24] Toscano L.T., Tavares R.L., Toscano L.T., da Silva C.S.O., de Almeida A.E.M., Biasoto A.C.T., Gonçalves M.d.C.R., Silva A.S. (2015). Potential ergogenic activity of grape juice in runners. Appl. Physiol. Nutr. Metab..

[25] Torregrosa-García A., Ávila-Gandía V., Luque-Rubia A.J., Abellán-Ruiz M.S., Querol-Calderón M., López-Román F.J. (2019). Pomegranate Extract Improves Maximal Performance of Trained Cyclists after an Exhausting Endurance Trial: A Randomised Controlled Trial. Nutrients.

[26] García-Merino J.Á., Moreno-Pérez D., de Lucas B., Montalvo-Lominchar M.G., Muñoz E., Sánchez L., Naclerio F., Herrera-Rocha K.M., Moreno-Jiménez M.R., Rocha-Guzmán N.E. (2020). Chronic flavanol-rich cocoa powder supplementation reduces body fat mass in endurance athletes by modifying the follistatin/myostatin ratio and leptin levels. Food Funct..

[27] Askari G., Ghiasvand R., Paknahad Z., Karimian J., Rabiee K., Sharifirad G., Feizi A. (2013). The effects of quercetin supplementation on body composition, exercise performance and muscle damage indices in athletes. Int. J. Prev. Med..

[28] Abbey E.L., Rankin J.W. (2011). Effect of quercetin supplementation on repeated-sprint performance, xanthine oxidase activity, and inflammation. Int. J. Sport Nutr. Exerc. Metab..

[29] Brandenburg J.P., Giles L.V. (2021). Blueberry supplementation reduces the blood lactate response to running in normobaric hypoxia but has no effect on performance in recreational runners. J. Int. Soc. Sports Nutr..

[30] Dean S., Braakhuis A., Paton C. (2009). The Effects of EGCG on Fat Oxidation and Endurance Performance in Male Cyclists. Int. J. Sport Nutr. Exerc. Metab..

[31] Decroix L., Tonoli C., Soares D.D., Descat A., Drittij-Reijnders M.-J., Weseler A.R., Bast A., Stahl W., Heyman E., Meeusen R. (2017). Acute cocoa Flavanols intake has minimal effects on exercise-induced oxidative stress and nitric oxide production in healthy cyclists: A randomized controlled trial. J. Int. Soc. Sports Nutr..

[32] Ruiz-Iglesias P., Gorgori-González A., Massot-Cladera M., Castell M., Pérez-Cano F.J. (2021). Does Flavonoid Consumption Improve Exercise Performance? Is It Related to Changes in the Immune System and Inflammatory Biomarkers? A Systematic Review of Clinical Studies since 2005. Nutrients.

[33] Albertus Y., Tucker R., Gibson A.S.C., Lambert E.V., Hampson D.B., Noakes T.D. (2005). Effect of Distance Feedback on Pacing Strategy and Perceived Exertion during Cycling. Med. Sci. Sports Exerc..

[34] Laursen P.B., Francis G.T., Abbiss C.R., Newton M.J., Nosaka K. (2007). Reliability of Time-to-Exhaustion versus Time-Trial Running Tests in Runners. Med. Sci. Sports Exerc..

[35] Van De Walle G.P., Vukovich M.D. (2018). The effect of nitrate supplementation on exercise tolerance and performance: A systematic review and meta-analysis. J. Strength Cond. Res..

[36] Liberati A., Altman D.G., Tetzlaff J., Mulrow C., Gøtzsche P.C., Ioannidis J.P., Clarke M., Devereaux P.J., Kleijnen J., Moher D. (2009). The PRISMA statement for reporting systematic reviews and meta-analyses of studies that evaluate health care interventions: Explanation and elaboration. Ann. Intern. Med..

[37] Cumpston M., Li T., Page M., Chandler J., Welch V., Higgins J.P., Thomas J. (2019). Updated guidance for trusted systematic reviews: A new edition of the Cochrane Handbook for Systematic Reviews of Interventions. Cochrane Database Syst. Rev..

[38] Higgins J., Green S., Ben Van Den A. (2020). Cochrane Handbook for Systematic Reviews of Interventions. Int. Coach. Psychol. Rev..

[39] Hozo I., Djulbegovic B., Clark O., Lyman G.H. (2005). Estimating the mean and variance from the median, range, and the size of a sample. BMC Med. Res. Methodol..

[40] LV H. (1985). Estimation of a single-effect size: Parametric and non-parametric methods. Statistical Methods for Meta-Analysis.

[41] Hedges L.V. (1981). Distribution theory for Glass’s estimator of effect size and related estimators. J. Educ. Stat..

[42] Andrade C. (2020). Mean difference, standardized mean difference (SMD), and their use in meta-analysis: As simple as it gets. J. Clin. Psychiatry.

[43] DerSimonian R., Laird N. (1986). Meta-analysis in clinical trials. Control. Clin. Trials.

[44] Nieman D.C., Williams A.S., Shanely R.A., Jin F., Mcanulty S.R., Triplett N.T., Austin M.D., Henson D.A. (2010). Quercetin’s Influence on Exercise Performance and Muscle Mitochondrial Biogenesis. Med. Sci. Sports Exerc..

[45] Egger M., Smith G.D., Schneider M., Minder C. (1997). Bias in meta-analysis detected by a simple, graphical test. BMJ.

[46] Bentley D.J., Dank S., Coupland R., Midgley A., Spence I. (2012). Acute Antioxidant Supplementation Improves Endurance Performance in Trained Athletes. Res. Sports Med..

[47] Bigelman K.A., Fan E.H., Chapman D.P., Freese E.C., Trilk J.L., Cureton K.J. (2010). Effects of Six Weeks of Quercetin Supplementation on Physical Performance in ROTC Cadets. Mil. Med..

[48] Howatson G., Snaith G.C., Kimble R., Cowper G., Keane K.M. (2022). Improved endurance running performance following haskap berry (*Lonicera caerulea* L.) ingestion. Nutrients.

[49] MacRae H.S.-H., Mefferd K.M. (2006). Dietary Antioxidant Supplementation Combined with Quercetin Improves Cycling Time Trial Performance. Int. J. Sport Nutr. Exerc. Metab..

[50] Martínez-Noguera F.J., Marín-Pagán C., Carlos-Vivas J., Rubio-Arias J.A., Alcaraz P.E. (2019). Acute Effects of Hesperidin in Oxidant/Antioxidant State Markers and Performance in Amateur Cyclists. Nutrients.

[51] Martínez-Noguera F.J., Marín-Pagán C., Carlos-Vivas J., Alcaraz P.E. (2020). Effects of 8 Weeks of 2S-Hesperidin Supplementation on Performance in Amateur Cyclists. Nutrients.

[52] McCormick R., Peeling P., Binnie M., Dawson B., Sim M. (2016). Effect of tart cherry juice on recovery and next day performance in well-trained Water Polo players. J. Int. Soc. Sports Nutr..

[53] Overdevest E., Wouters J.A., Wolfs K.H.M., Van Leeuwen J.J.M., Possemiers S. (2018). Citrus Flavonoid Supplementation Improves Exercise Performance in Trained Athletes. J. Sports Sci. Med..

[54] Perkins I.C., Vine S.A., Blacker S.D., Willems M.E.T. (2015). New Zealand Blackcurrant Extract Improves High-Intensity Intermittent Running. Int. J. Sport Nutr. Exerc. Metab..

[55] Seeley A.D., Jacobs K.A., Signorile J.F. (2019). Acute soy supplementation improves 20-km time trial performance, power, and speed. Med. Sci. Sport. Exerc..

[56] Sharp M.A., Hendrickson N.R., Staab J.S., McClung H.L., Nindl B.C., Michniak-Kohn B.B. (2012). Effects of Short-Term Quercetin Supplementation on Soldier Performance. J. Strength Cond. Res..

[57] Van Iersel L.E., Stevens Y.R., Conchillo J.M., Troost F.J. (2021). The effect of citrus flavonoid extract supplementation on anaerobic capacity in moderately trained athletes: A randomized controlled trial. J. Int. Soc. Sports Nutr..

[58] Yarahmadi M., Askari G., Kargarfard M., Ghiasvand R., Hoseini M., Mohamadi H., Asadi A. (2014). The effect of anthocyanin supplementation on body composition, exercise performance and muscle damage indices in athletes. Int. J. Prev. Med..

[59] Fairlie-Jones L., Davison K., Fromentin E., Hill A.M. (2017). The Effect of Anthocyanin-Rich Foods or Extracts on Vascular Function in Adults: A Systematic Review and Meta-Analysis of Randomised Controlled Trials. Nutrients.

[60] Hopkins W.G., Hawley J.A., Burke L.M. (1999). Design and analysis of research on sport performance enhancement. Med. Sci. Sports Exerc..

[61] Murase T., Haramizu S., Shimotoyodome A., Tokimitsu I., Hase T. (2006). Green tea extract improves running endurance in mice by stimulating lipid utilization during exercise. Am. J. Physiol. Integr. Comp. Physiol..

[62] Murase T., Haramizu S., Shimotoyodome A., Nagasawa A., Tokimitsu I. (2005). Green tea extract improves endurance capacity and increases muscle lipid oxidation in mice. Am. J. Physiol. Integr. Comp. Physiol..

[63] Wolfram S., Wang Y., Thielecke F. (2006). Anti-obesity effects of green tea: From bedside to bench. Mol. Nutr. Food Res..

[64] Green D.J., Maiorana A., O’Driscoll G., Taylor R. (2004). Effect of exercise training on endothelium-derived nitric oxide function in humans. J. Physiol..

[65] Newsholme P., De Bittencourt P.I.H., Hagan C.O., De Vito G., Murphy C., Krause M.S. (2010). Exercise and possible molecular mechanisms of protection from vascular disease and diabetes: The central role of ROS and nitric oxide. Clin. Sci..

